# Concepts of extracellular matrix remodelling in tumour progression and metastasis

**DOI:** 10.1038/s41467-020-18794-x

**Published:** 2020-10-09

**Authors:** Juliane Winkler, Abisola Abisoye-Ogunniyan, Kevin J. Metcalf, Zena Werb

**Affiliations:** Department of Anatomy, Helen Diller Family Comprehensive Cancer Center, University of California, San Francisco, CA 94143 USA

**Keywords:** Biological sciences, Cancer microenvironment

## Abstract

Tissues are dynamically shaped by bidirectional communication between resident cells and the extracellular matrix (ECM) through cell-matrix interactions and ECM remodelling. Tumours leverage ECM remodelling to create a microenvironment that promotes tumourigenesis and metastasis. In this review, we focus on how tumour and tumour-associated stromal cells deposit, biochemically and biophysically modify, and degrade tumour-associated ECM. These tumour-driven changes support tumour growth, increase migration of tumour cells, and remodel the ECM in distant organs to allow for metastatic progression. A better understanding of the underlying mechanisms of tumourigenic ECM remodelling is crucial for developing therapeutic treatments for patients.

## Abstract (web summary)

Tumors are more than cancer cells — the extracellular matrix is a protein structure that organizes all tissues and is altered in cancer. Here, the authors review recent progress in understanding how the cancer cells and tumor-associated stroma cells remodel the extracellular matrix to drive tumor growth and metastasis.

## Introduction

Cellular phenotypes and molecular functions are fundamentally dependent on signals from outside the cell such as the interactions with the extracellular matrix (ECM). The core matrisome is composed of ~300 unique matrix macromolecules and can be classified into collagens, proteoglycans (such as heparan sulphate proteoglycans, versican and hyaluronan) and glycoproteins (such as laminins, elastin, fibronectin and tenascins)^1^. These ECM components are modified post-translationally by an array of secreted remodelling enzymes, such as oxidases and proteases. In addition, the ECM binds soluble factors, such as growth factors and other ECM-associated proteins. Cell surface receptors interact with ECM components and ECM-bound factors to mediate cell adhesion and cell signalling thereby regulating processes as diverse as proliferation, differentiation, migration and apoptosis^2^. ECM can also demonstrate very different mechanical and topographical properties, which, importantly, can influence cell fate and function via different mechanosignalling routes^3^.

The ECM has two main forms, which differ in function, composition and location. The interstitial matrix forms porous three-dimensional networks around cells that interconnect cells in the stroma and can connect to the basement membrane, which is the other form of ECM structure. The interstitial matrix guarantees the structural integrity of tissues and organs but also modulates processes such as cell differentiation and migration. The protein composition of the interstitial matrix mainly includes collagens I, III, V, etc., fibronectin and elastin. Abundance and composition of the interstitial matrix vary between tissue types, between microenvironments within the same tissue and can be remodelled in response to force stress or trauma such as wound repair or tissue regeneration^4^. In cancer, remodelling of the interstitial ECM induces a broad range of biophysical and biochemical changes affecting cell signalling, ECM stiffness, cell migration and tumour progression^5^. In contrast, the basement membrane is a more stable, sheet-like, dense structure that lines the basal surface of, for example, epithelial and endothelial cells, surrounds muscle cells and adipocytes^6^, and separates tissues into different, well-organised compartments. The basement membrane consists mainly of collagen IV and laminins, which are interconnected through different network-bridging proteins such as nidogen and heparan sulphate proteoglycans (HSPGs)^7^. Binding of cells to the basement membrane is essential for establishing epithelial cell polarity and is crucial for many developmental processes and maintenance of tissue homoeostasis^8^. Remodelling of the basement membrane is required for cancer cells to invade stromal tissue and become a malignant tumour^9^.

Complex ECM remodelling processes, involving over 700 proteins^1^, change overall abundance, concentration, structure and organisation of individual ECM components, thereby affecting the three-dimensional spatial topology of the matrix around cells, its biochemical and biophysical properties and consequently its effect on cell fate. ECM remodelling is an essential and tightly regulated physiological process in development and in restoring tissue homoeostasis during wound repair^10^. However, it is not surprising that cells dysregulate this process in pathologic conditions such as inflammatory diseases, tissue fibrosis, and cancer^11^. Recent research highlights the importance of the tumour-mediated systemic aberrations of the ECM for the establishment of metastasis. In this review, we discuss remodelling mechanisms of extracellular matrices and the implications of these mechanisms during cancer development, and describe recent concepts of ECM remodelling shaping tissues for tumour cells to metastasise. Increasing understanding of these processes opens up the possibilities of therapeutic approaches to target the aberrant ECM and/or the underlying pathologic mechanisms of its remodelling and prevent malignancy.

## Mechanisms of tumourigenic ECM remodelling

**Fig. 1:**
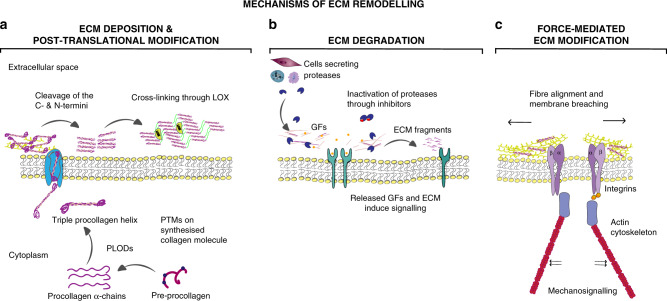
Mechanisms of ECM remodelling. **a** ECM deposition and modification: using collagen as an example, pre-procollagen is synthesised and translocated to the Golgi, where it becomes a procollagen α-chain. This procollagen molecule undergoes several post-translational modifications (PTMs) to modify its properties. The PTMs include glycosylation, pro-peptide alignment, disulphide bond formation and hydroxylation. Lysine hydroxylation of the procollagen chains by PLODs allows for spontaneous triple helix formation within the cell and secretion into the extracellular space. Here, the pro-peptides on the C- and N-terminal are cleaved by proteases creating collagen fibrils. For further collagen fibre assembly, collagens fibrils are cross-linked by LOX. **b** ECM degradation: proteases including MMPs cleave the ECM proteins, which releases matrix-bound growth factors (GFs) and cytokines, and ECM fragments, including matrikines and also remove barriers for cell migration. **c** Force-mediated ECM remodelling: integrin binding to the ECM molecules applies forces to ECM molecules. This can change the conformation of the ECM molecule, thereby exposing binding sites to support self-assembly into fibrils that induces fibre alignment. The mechanical force applied by the integrins in this modification process can also cause non-proteolytic breaching of the basement membrane that will allow cancer cell invasion.

Changes in the ECM are a result of different remodelling mechanisms that can be divided into four main processes: (1) ECM deposition, which changes the abundance and composition of ECM components, thereby affecting biochemical and mechanical ECM properties; (2) chemical modification at the post-translational level, which alters the biochemical properties and structural characteristics of the ECM (Fig. 1a); (3) proteolytic degradation, which releases bioactive ECM fragments and ECM-bound factors and may be required for the liberation of cellular constraints, such as migratory barriers (Fig. 1b); and (4) force-mediated physical remodelling, which affects ECM organisation by aligning ECM fibres and opening-up passages for cell migration (Fig. 1c).

**Fig. 2:**
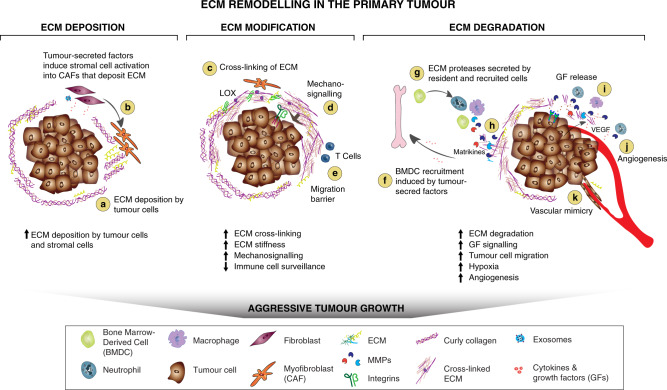
ECM remodelling in the primary tumour. **a**, **b** Tumour-derived factors activate stromal cells which differentiate into cancer-associated fibroblasts (CAFs) leading to the secretion and deposition of large amounts of ECM components along with the cancer cells. **c** ECM-modifying enzymes such as LOX expressed by tumour cells and CAFs cross-link and align collagen fibres, which increases matrix stiffness around the tumour, and **e** the formation of a physical barrier to evade immune surveillance by T-cells. **d** Increased matrix stiffness promotes the interaction between ECM components and cell-surface receptors on tumour cells that triggers mechanosignalling mediated by integrins. **f** To sustain a tumourigenic microenvironment, tumour cells and resident immune cells secrete cytokines, chemokines and growth factors (GFs), which differentiate and recruit bone marrow-derived cells (BMDCs). **g** The BMDCs, CAFs and tumour cells secrete ECM-degrading proteases, including MMPs, which are cell surface-bound (e.g., MT1-MMP) or secreted (e.g., MMP-9). **h** Proteolytic ECM degradation generates bioactive matrikines and **i** releases matrix-bound GFs. These factors induce pro-tumourigenic ECM signalling that promotes tumour proliferation, migration, invasion and angiogenesis. **j** These combined changes to the ECM create a hypoxic environment. Neutrophils secrete potent MMP-9 that degrades ECM and releases matrix-bound VEGF that forms a concentration gradient for new angiogenic sprouting. **k** Stimulated by dense ECM, the tumour cells may gain endothelial-like functions and mimic the vasculature that connects to blood vessels.

Tissue homoeostasis is dependent on the tight regulation between ECM deposition, modification, degradation and organisation that give rise to the biochemical and biophysical ECM properties. Changing single elements can turn over the delicate balance of ECM remodelling events. ECM alterations have implications on complex cellular signalling networks since ECM components serve as ligands for various cell surface receptors such as integrins, syndecans and receptor tyrosine kinases. Thus, it is not surprising that cancer cells and tumour-associated stromal cells modify all four ECM remodelling mechanisms, creating a cancer-supporting matrix that actively contributes to the pathology of the tumour (Fig. 2)^12^.

### Cellular deposition of tumourigenic ECM

In principle, ECM components can be produced and deposited by any cell. Tumour cells themselves show altered expression of ECM components, such as collagens I and III and ECM-modifying enzymes, such as lysyl oxidases (LOX) and LOX-like proteins (LOXL) (Fig. 2a)^12–15^. However, both in healthy tissue and tumours, the major producers of ECM in the interstitial matrix are fibroblasts, especially when activated and transformed to myofibroblasts; chondrocytes and osteoblasts are also significant, depending on tissue and context^16^. Myofibroblasts have a combined phenotypic characteristic of fibroblasts and smooth muscle cells, whereby they secrete ECM and exert contractile functions enabling mechanical alterations of the topology of the ECM, respectively. They can originate from different cell types and are mostly characterised by de novo expression of α-smooth muscle actin (α-SMA), although there are other markers of myofibroblasts in different conditions (reviewed in refs. ^16,17^). Activation of myofibroblasts is triggered by various pro-inflammatory factors^18^, most importantly transforming growth factor β (TGF-β)^19^. ECM remodelling by myofibroblasts is an essential physiologic process, for example in tissue regeneration and wound repair, which includes their inactivation after restoring homoeostasis. However, sustained inflammatory stimuli and subsequent TGF-β secretion by immune cells such as macrophages and tumour cells can generate deregulated, hyperproliferative and overactive myofibroblasts that are the major culprits in pathologic conditions such as fibrosis and cancer^20^.

Tumour cells primarily orchestrate the recruitment and activation of stromal cells that are the major depositors of ECM components in the tumour microenvironment (Fig. 2b) by the secretion of various pro-fibrotic growth factors and inflammatory factors such as TGF-α, TGF-β, fibroblast growth factor (FGF)-2, platelet-derived growth factor (PDGF) and epidermal growth factor (EGF)^21^. Recent evidence indicates that several of these stromal activation signals can be secreted in exosomes, which may impact the activation and differentiation to a myofibroblast phenotype, as exosomal TGF-β elevates FGF-2 production in fibroblasts compared to non-exosomal TGF-β^22^.

The tumour-derived activation factors induce the differentiation of stromal cells towards so-called cancer-associated fibroblasts (CAFs), which function as myofibroblasts and remodel the ECM to support tumour growth^17,20,23,24^. These activated stromal cells are of multiple origins, prominently including tissue-resident or bone marrow-derived fibroblasts^25^, but also, for example, adipocytes^26^. Interestingly, stress-induced adrenergic signalling in ovarian cancer cells can also activate CAFs to deposit elevated amounts of collagen that can be inhibited by β-blockers^27^. Furthermore, in aged tissue, stromal cells show alterations in ECM remodelling properties such as changes in the expression of matrix metalloproteinases (MMPs) and chemokines that support tumour development and metastasis^28^. In melanoma, aged fibroblasts express lower amounts of the ECM-modifying protein hyaluronan and proteoglycan link protein 1 (HAPLN1) that leads to reduced integrity of lymphatic vasculature and increased metastasis^29^. Changes in the tumour ECM can also further activate stromal cells in a feed-forward mechanism^30,31^.

ECM deposition by CAFs may depend on their spatial arrangement in the tumour. In pancreatic ductal adenocarcinoma (PDAC), CAFs in direct interaction with adjacent tumour cells show active TGF-β signalling and deposit collagen, whereas CAFs at a greater distance are also activated by the tumour cells, but are unresponsive to TGF-β, deposit HA and establish a tumourigenic, pro-inflammatory environment through the expression of cytokines such as IL-6, or antigen presentation^32–34^; this fosters the recruitment and activation of immune cells, which, as discussed below, contribute to the establishment of the cancer niche.

It is important to note that subpopulations of CAFs additionally support tumour growth through mechanisms independent of ECM remodelling, such as promoting cancer stemness or preventing cancer cell recognition by T-cells^35,36^. CAFs also may have tumour-suppressive functions such as restraining tumour vasculature or supporting immune surveillance, since the ablation of CAFs in murine models of PDAC led to a more aggressive tumour phenotype, which also correlates with patient data^37,38^. Hence, the heterogeneity, spatial distribution and potential plastic nature of CAFs need to be considered carefully in the context of targeting the stromal compartment of tumours.

### Changes in ECM composition in cancer

ECM components possess both tumour-suppressing and tumour-promoting properties. For example, depending on its molecular weight hyaluronan (HA) functions as a tumour suppressor or a tumour promoter (reviewed in Bohaumilitzky et al.^39^). The tumour-resistance of the longest-lived rodent, the naked mole-rat, involves the expression of a unique HA with high molecular mass (HMM-HA) as a major ECM component. HMM-HA signalling through the CD44 receptor activates the expression of key tumour suppressor genes^40^, leading to a hypersensitive cell-cycle arrest, a common mechanism of tumour suppression^41^. In contrast, high levels of HA, and in particular small HA oligosaccharides (LMM-HA), are associated with poor prognosis in several tumours such as colorectal, breast and prostate cancer^42–46^. Here, dysregulated HA synthetase and HA-degrading hyaluronidase lead to the accumulation of LMM-HA^42,47^. LMM-HA directly interacts with cell surface receptors regulating pro-tumourigenic signalling cascades^48^, including glycolysis, the main source of energy in tumours, and promotes migration^49^. LMM-HA signalling through CD44 also increases the resistance to cellular stress and thereby may promote tumour development^50^.

The most common tumourigenic alteration of ECM homoeostasis is an increased deposition of fibrillar collagen^13,51,52^, which has direct tumour-promoting properties. Additionally, increased deposition of major ECM components including fibronectin, HA and tenascin C into the interstitial matrix results in a fibrotic phenotype, termed desmoplasia, which is similar to the alterations observed during organ fibrosis. Desmoplasia is a key characteristic of various cancers such as breast cancer and PDAC and is associated with poor prognosis^53–55^. For example, in a murine breast cancer model, the increased deposition of collagen I results in increased tumour formation and development of metastasis, directly linking ECM remodelling with aggressive tumour progression in vivo^56^. Furthermore, collagen V has been associated with altered mitogenic signalling in breast cancer. It can enhance the co-receptor ability of the surface proteoglycan, glypican-1 (GPC1), to modify FGF-2 signalling leading to increased cell proliferation^56,57^.

Analysing the complex global changes in the ECM may be beneficial for early cancer detection^58^. Unique ECM signatures that are characteristic of some tumours change during tumour progression and are predictive of clinical outcomes^59–61^. The combined pathologic alterations of the ECM provide a protein fingerprint by the release of cancer-specific ECM fragments into the circulation that may have diagnostic implications as it was shown for lung, ovarian, breast and colorectal cancer^62–64^. Moreover, even before tumour development, increased deposition of collagen I and proteoglycans lead to increased breast density, which is the greatest independent risk factor for the development of breast cancer (Box 1)^65–67^.

Box 1 breast density and cancer risk In the breast, mammographic density is indicative of a higher risk for breast cancer development, which is used for preventive screening^276,277^. High mammographic density is mediated by an increased ECM (e.g., collagen and proteoglycan) deposition and further cross-linking and alignment events which all together also result in stiffer breast tissue. A complex interplay of hormonal, inflammatory and environmental factors may all influence breast density^276,277^. So far, the detailed underlying mechanisms that trigger these ECM changes and how breast density increases breast cancer risk^278^ are unclear.

Breast cancer risk is also influenced by the fundamental changes that the mammary gland undergoes during different reproductive states, which are characterised by profound ECM remodelling processes^279^. Whether or not pregnancy protects or promotes breast cancer is still controversial and largely depends on the age of the woman during her first birth^280^. During pregnancy, the breast epithelium and tissue matrix are intensively remodelled to prepare for lactation. After nursing, the lactating glands and surrounding breast stroma are remodelled to its pre-pregnancy-like state in a process called involution. These remodelling processes are exceptionally rapid and comprehensive. Each state is characterised by a massive change in ECM abundance and composition. Following pregnancy, the breast retains a more developed lobular architecture^281^, which may also affect the proportion of epithelial cell types in the breast^282^. Furthermore, even the fully regressed mammary gland shows a higher abundance of ECM components, in particular collagen I, compared to the pre-pregnancy (nulliparous) state^283^. During involution, massive ECM proteolysis mediated by various upregulated MMPs leads to the disruption and degradation of the basement membrane, thereby detaching epithelial cells, which triggers cell death of unnecessary secretory mammary epithelium. High MMP activity also leads to the release of bioactive matrix proteins, matrikines, and growth factors and the recruitment of ECM-clearing immune cells^284^. Interestingly, ECM remodelling during involution shows high similarities to processes during inflammation and wound healing, which was also shown to provide a bonafide environment for cancer development through pro-inflammatory TGF-β^285^. Together, these inflammatory conditions and the loss of the barrier function of the basement membrane temporally create a pro-tumourigenic environment that may increase the risk for more aggressive breast cancer^280^.

### Changes in ECM modification and organisation in cancer

Once synthesised, the ECM proteins undergo post-translational modifications (PTMs) inside and outside the cell, which enhances their complexity and three-dimensional organisation (Table 1, reviewed by Yuzhalin et al.^68^). Post-translational modifications of ECM components affect matrix interactions with other molecules and cellular receptors, localisation within the tissue and ECM degradation^69^. We elaborate below how several key post-translational modifications are altered in a cancerous environment.

**Table 1:** Post-translational modifications of ECM and ECM-associated molecules and their implications in cancer.

Post-translational modification	Protein targets	Effectors	Cancer types	Associated changes in cancer
Hydroxylation	Collagen	Procollagen-lysine 1, 2-oxoglutarate 5-dioxygenases (PLODs)	Many types, including bladder^254^, lung^255^, and breast cancers^256^, and renal cell carcinoma^257^	Increased expression of PLODs in cancer and stromal cells increases collagen cross-linking and correlates with poor survival. PLOD2 is induced by hypoxic conditions^71^.
	Collagen	Prolyl-4-hydroxylase subunit alpha-1 (P4HA)	Breast cancer	Inhibition of collagen hydroxylation increases breast cancer-derived lung metastasis in mice^258^.
Cross-linking	Collagen, elastin	Lysyl oxidase (LOX) and lysyl oxidase homologues (LOXLs)	Many types, including colorectal, pancreatic, breast, lung, and prostate^259^	Overexpression of LOX and LOXLs increases fibrosis and ECM stiffness and promotes tumourigenesis and metastasis^259^.
	Fibronectin, HSPB, fibrinogen, collagen VI	Transglutaminases	Many types, including PDAC, glioblastoma, melanoma^260^, and breast cancer^261^	Overexpression of TG2 in cancer cells and metastatic cancer cells^262^ increases ECM cross-linking, affects mechanical properties and cell–matrix signalling^68^.
Glycosylation	Fibronectin	Glycosyltransferases and glycosidases	Urothelial carcinoma^263^	Increased fibronectin glycosylation is correlated with increased invasiveness of urothelial carcinoma^263^ and enhanced EMT in human prostate cancer cell lines^264^.
	αvβ3 and αvβ6 integrins	Glycosyltransferases and glycosidases	Breast cancer	Inhibition of glycosylation increases the invasion properties of metastatic cells^265^.
Phosphorylation	Fibronectin	Casein kinase II-like protein kinase	Many types^266^	Phosphorylated fibronectin increases mechanical cell functions and cell traction forces for attachment^266^ and occurs at growth factor binding sites^267^.
	Many targets including MMPs and laminin A1	Vertebrate lonesome kinase (VLK) and extracellular serine/threonine protein kinase (FAM20C)	Currently poorly understood^268^	Extracellular kinases phosphorylate ECM and ECM-associated components, which can potentially alter downstream kinase signalling pathways^268,269^.
Sulphation	Glycosaminoglycans (GAGs)	Sulfotransferases and heparanase	Many types, including breast, ovarian, colorectal, prostate, and gastric cancers^270^	Changes in the degree of sulphation and/or the pattern in chondroitin sulphates and heparan sulphates in glycosaminoglycans are associated with different cancers by changing cell–cell and cell–matrix signalling^270–272^.
Citrullination	Collagen	Protein arginine deiminase 4 (PAD4)	Liver metastases of colorectal cancer	Promotes liver metastases of colorectal cancer^215^.
Isomerization	C-terminal telopeptide of type I collagen	Non-enzymatic	Breast and prostate cancer	High levels of non-isomerised C-telopeptide of collagen I (α-CTX-I) are indicative of high ECM turnover in bone metastases^220^.
Carbamylation	Collagen	Non-enzymatic	Currently poorly understood	Increased carbamylation decreases stability of collagen I triple helices and affects their degradation by MMPs^273^. Carbamylated collagen type I affects cancer cell migration^274^.
Glycation	Collagen	Non-enzymatic	Currently poorly understood	Glycated collagen type I affects cancer cell migration^274^.

For example, collagen chains are synthesised as long precursors (procollagens) that are further modified by various post-translational modifications, for example, glycosylation of their lysine and hydroxylysine residues by addition of galactose and glucose; and hydroxylation of lysine residues through lysyl hydroxylases (encoded by procollagen-lysine 2-oxoglutarate 5-dioxygenase (PLOD) genes)^70–72^. These modified procollagens form triple helices and are further processed extracellularly by proteases to create collagen fibrils. Additionally, collagen fibrils are covalently cross-linked by extracellular LOX and LOXLs, which is essential for correct collagen fibre assembly and increases tensile strength and stiffness (Fig. 2c)^73–75^ (reviewed by Mouw and Weaver^4^). Tissue transglutaminase 2 (TG2) cross-links ECM molecules such as fibronectin, HSPG, fibrinogen and collagen IV^68^ by the transamidation of glutamine residues to the amino group of a lysine residue of another protein chain. This transamidation process results in the formation of covalent N-γ-glutaminyl-ɛ-lysyl-isopeptide bonds, which are resistant to proteolytic degradation^69^.

Alterations of these post-translational modifications in tumourigenesis cause morphological changes in the ECM architecture that are indicative of tumour progression and influence tumour cell motility^76,77^. In normal soft tissue, collagen fibres in the interstitial matrix are curly and oriented parallel to the layer of epithelium^5^. A high deposition of these curly collagen I fibres in non-tumourous breast tissue can be protective against cancer development through the activation of tumour-suppressive phenotypes such as upregulation of genes encoding components of cell–cell junctions, and downregulation of mesenchymal-specific and metalloproteinase-encoding genes^78^. However, in breast cancer, collagen fibres in proximity to the tumour boundary are linearised, perpendicularly oriented and supportive of invasive tumour growth^15,79^. Further, PLODs, LOXs and TG2 are frequently overexpressed in cancer^68^ that, together with force-mediated ECM remodelling events, cause increased cross-linking and linearization of ECM molecules.

The majority of extracellular and transmembrane proteins are glycosylated. Transmembrane glycoproteins function as receptors for ECM molecules; for example, CD44 is heavily glycosylated and its ECM ligand HA is a glycosaminoglycan. Other glycosylated ECM molecules include HSPGs that bind growth factors and modify receptor tyrosine kinase signalling^80^. Membrane-anchored glycoproteins, together with glycolipids and polysaccharides, form a complex and heterogenous structure on the cell surface, termed the glycocalyx, that interacts with the ECM^81^. The glycocalyx mediates cell–ECM, cell–cell interactions (for example, during immune cell surveillance^82,83^), binding of growth factors, and mechanoresponses^84^. Interestingly, glycosylation patterns of surface proteins frequently change in cancer and may foster pathological cell behaviours^84–86^. Tumour cells upregulate bulky glycoproteins on the cell surface. The resulting bulky glycocalyx can apply tension to ECM-bound integrins, which are important receptors for ECM components, causing integrin clustering and thereby increasing pro-tumourigenic integrin signalling^84^.

Modifications of ECM components also affect their capacity of binding to growth factors. Extracellular HSPGs regulate the activity of various receptor tyrosine kinase signalling pathways by their ability to store various growth factors, the release of which is regulated by extracellular modification enzymes such as endosulphatases (Sulfs). Sulfs, which are dysregulated in many cancers, modify the sulphation pattern of HSPGs and affect the binding of growth factors. Sulf1 can act as a tumour suppressor, whereas Sulf2 activates pro-tumourigenic signalling pathways^87–89^. Sulf1 is downregulated in ovarian cancer and a subset of hepatocellular carcinoma, which prevents Sulf1-mediated inactivation of receptor tyrosine kinase signalling^90,91^. However, Sulf2 supports Wnt and PDGFRα signalling in vitro^92,93^. Since Sulfs act extracellularly, it is likely that stromal cells contribute to Sulf levels in the tumour ECM. Although both Sulf isotypes show similar substrate specificity, conflicting studies, in particular for Sulf1, suggest that the functional consequences of HSPG modification may depend on experimental conditions (in vitro vs. in vivo), context and tumour or tumour subtype.

### Degradation of the tumourigenic ECM

The ECM is cleaved and degraded by target-specific proteases such as MMPs, disintegrin and metalloproteinases (ADAMs), disintegrin and metalloproteinases with thrombospondin motifs (ADAMTS), and proteases that specifically cleave at serine, cysteine and threonine residues (reviewed in refs. ^10,94^). These proteases are secreted primarily by stromal cells, including recruited bone marrow-derived cells, as well as by cancer cells (Fig. 2f–g). Several proteases are regulated by an activation cascade by which different proteases that are secreted as inactive pro-forms or bound to the ECM are released and activated by other proteases^9^. The activity of proteases is counterbalanced by protease inhibitors (e.g., tissue inhibitor of metalloproteinases, TIMPs)^95^. The substrates of the different proteases are still not completely understood. Recent advances in the sensitivity of proteomics techniques allow targeted degradomics studies to identify precise proteolytic cleavage targets from protease-substrate interactions^96,97^. These studies revealed that proteases cleave different substrates when they are bound to the cell membrane compared to their soluble form. ADAM10 and 17 are membrane-anchored and their ectodomains can be released by ADAM8. The soluble ectodomains show different target specificity^98^.

Proteolytic degradation of ECM components can be both pro-tumourigenic^73,94^ and anti-tumourigenic^99,100^. MMP inhibitors, designed to block ECM remodelling and decrease invasion, have thus far underperformed in clinical trials, likely due to the pleiotropic activities of MMPs^99^. As an example, MMP-8 has pro-tumour functions, where overexpression of MMP-8 in cancer cells is correlated with decreased survival in patients with ovarian cancer^101^ and with hepatocellular carcinoma^102^. However, MMP-8 also has anti-tumour activity, as MMP-8 decreased cancer cell invasion in vitro and low expression of MMP-8 was correlated with decreased survival in patients with oral tongue squamous cell carcinoma^103^. Further, germline deletion of MMP-8 in mice increased susceptibility to chemically induced skin tumours, and bone marrow transplants of MMP-8-expressing neutrophils restored tumour protection^104^. These conflicting results highlight that context-dependent effects of ECM-degrading enzymes on tumour growth and invasion.

Tumour and stromal cells express increased levels of ECM-degrading proteases that have multiple functions during tumour progression^9^. First, proteolytic degradation of ECM components allows progressive destruction of the normal ECM and its replacement with tumour-derived ECM. Second, ECM degradation is an important driver of cancer cell motility (see below). Third, ECM-binding of soluble signalling molecules such as growth factors makes them insoluble and inactive and action of proteases liberate them. Fibronectin, for example, binds insulin-like growth factor binding-protein-3, FGF-2 and VEGF-A with high affinity^105^. The enhanced protease activity in tumours causes subsequent ECM degradation that releases the ECM-bound growth factors and thereby increases their bioavailability (Fig. 2i).

Last, the cleavage of long ECM components produces bioactive, shorter fragments with distinct functions that can be pro- or anti-tumourigenic compared to the full-length ECM component. These fragments often contain structures similar to chemokines or cytokines and are therefore termed matrikines (Fig. 2h, reviewed in refs. ^106,107^). Matrikines play an important role in angiogenesis and balance the angiogenic switch through promoting and inhibiting properties (reviewed in ref. ^108^). Anti-angiogenic functions are described for several NC1 domains of basement membrane collagens^109,110^, such as the matrikine endostatin^108,111^. A specific domain of the α3 chain of type IV collagen has anti-tumour effects through the inhibition of the expression of membrane-type 1-matrix metalloproteinase (MT1-MMP or MMP14) and integrin β3, which leads to impaired migratory abilities of tumour cells^112^. On the other hand, various elastin-derived matrikines, such as Val-Gly-Val-Ala-Pro-Gly (VGVAPG) or Ala-Gly-Val-Pro-Gly-Leu-Gly-Val-Gly (AGVPGLGVG) promote tumour progression^113^. These ECM fragments are products of the degradation of elastin through different proteolytic enzymes (elastases)^114^ and MMPs^115^. These matrikines can, in turn, also induce MMP expression and activation, including MT1-MMP and MMP-2, which would explain their tumour-promoting properties^116^.

Overall, the combined pro-tumourigenic functions of ECM degradation highlight the importance of proteases for tumour progression. Protease activity can be measured in real-time using Proteolytic Activity Matrix Analysis (PrAMA). Cleavage patterns of FRET-based polypeptides thereby reflect specific enzyme activities when compared to purified proteases as standard. This method allows the detection of enhanced protease activity in patient samples such as cerebrospinal fluid, which is indicative of neoplastic meningitis and metastasis to the brain^117^.

It is important to note that, in addition to proteolysis of ECM, MMPs have significant non-proteolytic functions that can alter tumour progression. For example, MMP-3 binds and inhibits noncanonical Wnt5b, resulting in increased canonical Wnt signalling^9^, and MT1-MMP promotes basal extrusion during epithelial-to-mesenchymal transition (EMT) and basal mitosis noncatalytically^118^.

## Impact of tumourigenic ECM remodelling

Remodelling of the tumour ECM impacts every stage of tumourigenesis. In this section we describe these effects on the development of a primary tumour, including how a tumourigenic ECM induces pro-tumour signalling in cancer cells, helps to create an immunosuppressive environment and further supports the progression of the tumour to grow, invade stromal tissue and promote vascularisation.

### Tumourigenic ECM remodelling rewires signalling

ECM components bind to integrins which are heterodimeric transmembrane receptors with bidirectional signalling that couple them to the actin cytoskeleton within the cell. Force-mediated ECM-integrin interaction induces various downstream signalling events, known as mechanosignalling (Fig. 2d). Repeated adhesion and de-adhesion of cells to the ECM substrate via integrins and the alteration of actin cytoskeletal reorganisation mediates cellular processes such as cell migration^119,120^. Syndecans are cell surface-bound HSPGs that interact with integrins to support focal adhesion formation and migration^80^. Integrin-mediated signalling includes pro-survival and pro-apoptotic pathways and their crosstalk with growth factor receptors mediates cellular signalling (reviewed in refs. ^121–123^).

As a result of the alterations in the ECM, tumours often are stiffer than normal tissue, which can be applied in clinical diagnosis^124,125^. The stiff matrix of the tumour leads to an altered mechanosignalling. High ECM deposition and increased stiffness combined with integrin overexpression in various cancers^126^ trigger tumour promotion (reviewed in Desgrosellier and Cheresh^121^).

As mentioned above, tumour-secreted TGF-β activates the differentiation of stromal cells into ECM-remodelling CAFs. Additionally, TGF-β secreted by CAFs can induce STAT3 signalling in tumour cells^127^. However, in PDAC, TGF-β loss in tumour cells is associated with increased desmoplasia and enhanced matrix tension. The pronounced desmoplasia triggers β1-integrin mechanosignalling that promotes tumour progression through STAT3 activation^128^. These observations show the complexity of TGF-β signalling in tumour and stromal cells, which can have pro- and anti-tumourigenic functions depending on the context.

Collagens also bind to unique transmembrane receptor tyrosine kinases, known as Discoidin Domain Receptors (DDR1 and DDR2). They induce cellular signalling in conjunction with other receptors such as integrins and Notch^129^. The high abundance of collagen in the tumour microenvironment triggers the activation of the receptor tyrosine kinase DDR receptors. DDR1 expressed by gastric cancer cells supports invasion and tumour metastasis^130^. In breast cancer, the interaction of collagen I with DDR1 expressed by tumour cells leads to the induction of stemness-like signalling through the activation of STAT3, which is crucial to mediate the metastatic outgrowth^131^. Surprisingly, DDR1 deletion in a breast cancer mouse model increases ECM deposition and promotes tumour aggressiveness and metastasis^132^. DDR2 deficiency in hepatic stellate cells triggers differentiation of CAFs and fosters the establishment of a metastatic niche^133^. These studies suggest that DDR1 and DDR2 are expressed by tumour and stromal cells and depending on receptor isotype, cell type and context can either support or suppress tumour development^129,132^.

### Tumour-immune cell crosstalk in ECM remodelling

The high ECM remodelling activity mediated by tumour cells and CAFs contributes to an inflammatory tumour environment^134^. Components of the ECM can function directly as inflammatory stimuli, known as danger-associated molecular patterns (DAMPs)^135^ that induce immune responses through the interactions with pattern recognition receptors (PRRs) expressed by immune cells^136^. ECM-degrading proteases liberate these pro-inflammatory ECM components such as LMM-HA and biglycan, as well as matrix-bound growth factors and cytokines^136,137^. Biglycan, for example, activates PRRs including Toll-like receptor 4 (TLR4) and TLR2 expressed by macrophages that drive the expression of TNF-α and macrophage inflammatory protein–2 (MIP-2/CXCL2)^138^. Matrikines may also serve as DAMPs in tumours. Versican is degraded by ADAMTS proteases to reveal a bioactive fragment called versikine^139^. Increased intratumoural levels of versikine leads to increased tumour infiltration of CD8^+^ T-cells in colorectal cancer^140^ and myeloma^141^. Increased CD8^+^ T-cell infiltration results from versikine-induced expression of the transcription factor Interferon Regulatory Factor 8 (IRF8) in macrophages and enhances generation of CD103^+^ CD11c^hi^MHCII^hi^ conventional dendritic cells (cDC) in a mouse model^140^.

Bone marrow-derived cells, such as tumour-associated macrophages (TAMs) and tumour-associated neutrophils (TANs), are important sources for ECM remodelling proteases in the primary tumour environment (Fig. 2g) and metastatic sites and promote tumour angiogenesis. Neutrophils are recruited to the tumour site by hypoxic conditions^142^ and constitute the major source of angiogenesis-inducing MMP-9 (Fig. 2j)^143^. This neutrophil-derived MMP-9 is particularly potent because it is not complexed with the MMP inhibitor, TIMP-1, unlike MMP-9 molecules secreted by other cell types^144^. TAMs sustain a pro-tumourigenic environment, predominately when polarised to a M2-like phenotype. M2-like TAMs induce proteolytic clearance of interstitial collagen through upregulated MMP expression, including pro-angiogenic TIMP-1-free MMP-9 similar to neutrophils, accompanied by increased endocytosis and lysosomal degradation of collagen^145,146^. In addition to ECM degradation, in a colorectal cancer model, TAMs contribute to ECM deposition. Interestingly, this study found that here TAMs are the major cell type to upregulate synthesis and assembly of collagens, specifically collagen types I, VI and XIV, and induce deposition, cross-linking and linearization of these collagen fibres near invasive tumour cells^147^. These data add to growing evidence that immune cells contribute to ECM deposition, as macrophages also deposit the glycoprotein osteonectin, which promotes stromal invasion in a mouse model of breast cancer^148^.

### ECM remodelling supports tumour cell migration and invasion

The altered physical properties of the stiff tumour ECM promote cell migration and invasion into the stromal tissue (reviewed in Kai et al.^12^). LOX-mediated collagen cross-linking stiffens the ECM in a murine breast cancer model, which triggers an integrin-dependent invasive phenotype (Fig. 2c)^15^. Additionally, collagen cross-linking mediated by PLOD2 expressed by CAFs in a murine lung adenocarcinoma model, increases tumour invasiveness^149^. However, the organisation and composition of the ECM, rather than density and stiffness alone, is crucial for tumour cell motility and tumour progression. For example, non-invasive breast tumour cells cultured in Matrigel, which contains non-fibrillar collagen IV, gain the potential for collective invasion when their culture matrix is changed to fibrillar collagen I, potentially due to fewer interactions with cell adhesion receptors, which anchor the cells to collagen IV-rich basement membranes^150,151^.

**Fig. 3:**
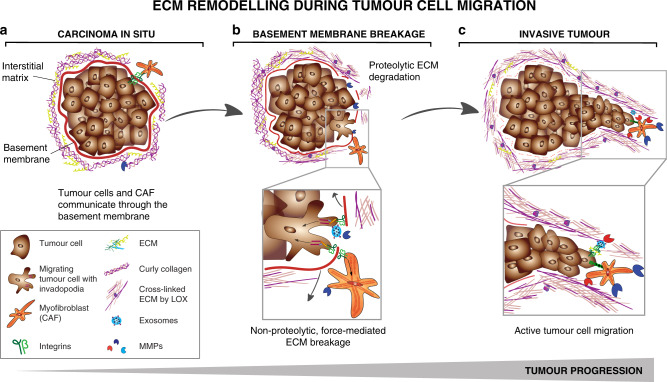
ECM remodelling during tumour cell migration. **a** In carcinoma in situ, tumour cells are restricted from migrating and invading into the surrounding tissue by the intact basement membrane. Tumour cells and stroma cells may be physically connected through the basement membrane using integrins. ECM in the interstitial matrix is curly. **b** Basement membrane breakage can be achieved through proteolytic ECM degradation by proteases secreted by tumour cells and activated stroma cells (top) and through non-proteolytic, force-mediated ECM remodelling (lower box). Integrins expressed on invadopodia, an invasive actin-rich cell structure on tumour and cancer-associated fibroblasts (CAFs), bind to ECM molecules and couple them intracellularly to contractile structures. This process pulls the ECM molecules apart and applies force to the basement membrane facilitating non-proteolytic basement membrane breaching. **c** In invasive tumours, the basement membrane is mostly degraded. CAFs and tumour cells secrete LOX to cross-link collagen fibres. Increased cross-linking and force-mediated ECM remodelling creates linearised ECM in the tumour-surrounding interstitial matrix. Tumour cells migrate along regions with dense, aligned collagen fibres forming migratory tracks for efficient cell migration. (lower box) Membrane-bound proteases expressed on tumour cells and CAFs, such as MT1-MMP localised on invadopodia, degrades the collagen migration barriers. Exosomes contain additional proteases to clear the ECM for tumour cell migration and are released into the interstitial matrix. Integrins on the surface of secreted exosomes bind to fibronectin, which functions as a bridging molecule connecting integrins expressed on tumour cells thereby promoting the formation of invadopodia and tumour cell migration. CAFs can function as leader cells for directed tumour cell migration and are connected to tumour cells through E-cadherin/N-cadherin adhesions.

During early solid tumour development, at the stage of carcinoma in situ, neoplastic cells are separated from the surrounding stromal cells by a basement membrane creating a physical barrier that prevents tumour cell dissemination (Fig. 3a). The tumour becomes malignant when the tumour cells break through the basement membrane into the interstitial space and invade the surrounding tissues – processes that are accompanied by various ECM remodelling events^41^.

Proteolytic degradation is important for breaching the basement membrane, although invasion through the basement membrane involves both proteolysis-dependent and -independent mechanisms (Fig. 3b)^152–154^. In vivo, breast tumour cells break through the basement membrane, mediated by membrane-anchored proteases, predominantly MT1-MMP^155–157^. Tumour cells and CAFs may be physically connected bridged by the layer of the basement membrane. Using their pronounced contractile properties CAFs can apply physical forces on the basement membrane and widen the pre-existing holes thus promoting tumour cell invasion in a MMP-independent manner^158^.

Proteolysis of ECM components is crucial for the active movement of cells through the interstitial matrix as it opens migratory tracks and reduces mechanical stress on the migrating cells^76,159,160^. T-cells and dendritic cells migrate towards chemoattractants along collagen type I fibres, which are enriched in the tumour environment^160,161^. However, as T-cell infiltration is non-proteolytic, they cannot migrate through tumour-associated dense collagen and fibronectin regions, and hence are excluded from the tumour and accumulate in the stroma (Fig. 2e)^162,163^. Also, age-related ECM alterations, such as specific cross-linking events, prevent sufficient T-cell movement^164,165^. Immunodeficiency due to decreased T-cell infiltration and consequent T-cell death may be attributed to the mechanical stress on the cytoplasm and nucleus of these T-cells during their passage through stiff ECM (reviewed by Moreau et al.^164^). Although under debate, these ECM migration barriers largely impair immune surveillance of tumours and have been associated with poor clinical outcome^163,166^.

In regions of dense collagen fibres in the tumour environment, pericellular proteolysis of the interstitial matrix is important for active cell migration and controlled by MMPs, particularly MT1-MMP on tumour cells and CAFs, which degrade the collagen migration barriers^167–169^. MT1-MMP is usually localised on invasive actin-rich cell structures called invadopodia^170^. There is now substantial evidence that these processes are supported by exosomes. Exosomes have been shown to promote maturation of invadopodia and ECM degradation via the extracellular delivery of MT1-MMP and other proteinases^171^. Additionally, integrins on the surface of secreted exosomes bind to fibronectin which thereby act as bridging molecules connecting integrins expressed on tumour cells. The local secretion of exosomes may, therefore, induce cellular integrin clustering that triggers adhesion assembly and result in more effective and directed cell migration^172^.

Owing to their high ECM-remodelling capacity, CAFs may serve as leader cells for tumour cell migration (Fig. 3c)^20^. They are positioned in front of invading cancer cells and clear the interstitial matrix through proteolytic- and force-mediated remodelling processes. CAFs interact with collagen-rich ECM through the integrins α3 and α5, and Rho-mediated regulation of myosin light chain activity. These interactions apply force to the ECM thereby aligning the collagen fibres^173,174^. Additionally, fibronectin binding to integrins, such as integrins αvβ3 and α5β1, on CAFs allows receptor clustering, which applies force on fibronectin dimers changing their conformation and exposing binding sites to support self-assembly into fibrils^4^. The force-mediated fibronectin assembly creates anisotropic fibre orientation and can open gaps in the matrix that allow cell migration^175^. Collagen linearization can also be mediated by tumour-secreted factors such as the WNT1 inducible signalling pathway gene (WISP1)^176^, which is triggered by TGF-β.

These processes create stable migratory tracks that are used for the efficient, proteolysis-independent collective invasion of the tumour cells that follow. Tumour cell migration is also actively guided by CAFs through force triggered E-cadherin/N-cadherin adhesion^177^. The specific architecture of these collagen tracks guides tumour cells to travel longer distances along the aligned collagen fibres by forming fewer but longer protrusions or to use amoeboid blebbing for optimal migration. The aligned matrix thereby supports, in particular, the directed migration of small, plastic tumour cells with stem-like characteristics^178–180^. This may explain why the signature of highly aligned collagen fibres is associated with poor prognosis^79^.

Overall, the physical properties of the tumour ECM together with its composition, density and the specific architecture of aligned fibres provide optimal migratory conditions for effective tumour cell dissemination and promote aggressive tumour phenotypes.

### ECM remodelling is fueled by hypoxia and promotes vascularisation

During tumour progression, rapid growth leads to a tissue architecture with tumour cells located distant from blood vessels with diffusion-limited oxygen and nutrient supply (reviewed in Semenza^181^). Hypoxia triggers cellular signalling mediated by the hypoxia-inducible transcription factor (HIF‐1). HIF-1 also promotes ECM remodelling through the regulation of various MMPs, for example, an upregulation of MMP-2, MMP-9 and MT1-MMP^182,183^, and activates collagen fibre alignment via the induction of various collagen-modifying enzymes, like prolyl-4-hydroxylases (P4HA1 and P4HA2), PLOD1 and PLOD2, LOX and LOX-like proteins (LOXL1, LOXL2 and LOXL4)^184–186^ in CAFs (reviewed in Gilkes et al.^70^), and additionally upregulates integrins, thereby contributing to enhanced pro-tumourigenic mechanosignalling^187^. Deposition of collagen I by mesothelial cells is also enriched by a hypoxic tumour-mesothelial niche, which promotes the metastasis of ovarian cancer^188^.

The state of hypoxia is a key driver for angiogenesis. HIF-1 expression in hypoxic tumour cells induces the angiogenic switch through the upregulation and activation of pro-angiogenic proteins such as VEGF to recruit endothelial cells and build new vessels^189^. To form functional new vessels endothelial tip cells rely on an upregulation of collagens and ECM-modifying enzymes such as LOX and PLOD proteins which was shown across different human tumour types^190^. VEGF secreted by cancer and stromal cells is bound to the ECM^191^ but is released through proteolytic cleavage^192,193^. HIF-1 expression induces immune cell recruitment, which significantly contributes to high concentrations of active MMP-9 in the ECM (Fig. 2j)^142^. The simultaneous HIF-1-induced upregulation of VEGF and increased MMP-9 levels lead to a high concentration of soluble VEGF in the tumour tissue driving angiogenesis and invasiveness. However, different ECM components promote and/or inhibit angiogenesis, which results in a disrupted tumour vasculature (reviewed in Sottile^108^). For example, periostin has pro-angiogenic functions^194^; tenascin-C and fibronectin have both pro- and anti-angiogenic functions depending on context^195^ (reviewed in Obberghen-Schilling^196^), and thrombospondin-1 inhibits MMP-9 activation and angiogenesis and thereby suppresses spontaneous tumour development^197^ (reviewed in Lawler and Lawler^198^).

The architecture of dense collagen in tumours also triggers the formation of interconnected networks of cancer cells. These tumour cells form ECM-rich vasculature-like structures, in a process called vasculogenic mimicry, that supply the tumour with blood without inducing angiogenesis (Fig. 2k). A specific, dense collagen architecture with small pores and short fibres causes an up-regulation of integrin-β1 and the differentiation of tumour cells towards an endothelial phenotype characterised by the expression of collagen type IV α1 chain and serine peptidase inhibitor Serpin E2, which have anticoagulant properties and are important for blood perfusion^150,199^. Vasculogenic mimicry of melanoma cells requires matrix remodelling through the expression of MMP-2, MT1-MMP and the deposition and cleavage of a specific laminin chain (γ2 chain of laminin 332)^200^. Interestingly, tumour-associated macrophages can also form vascular-like networks^201^. Besides providing an adequate blood supply for the tumour, vasculogenic mimicry is associated with an aggressive tumour phenotype and promotes metastasis^199^. Further research is needed to elucidate the underlying mechanisms of how the biochemical and biophysical properties of the ECM induce phenotypical changes in tumour cells and lead to formation of vascular-like structures.

Taken together, the characteristics of a tumour microenvironment maintains and promotes itself through complex interdependent pathways that lead to enhanced angiogenesis and invasiveness with the ultimate goal of metastasising to a secondary organ.

## ECM remodelling during metastasis

**Fig. 4:**
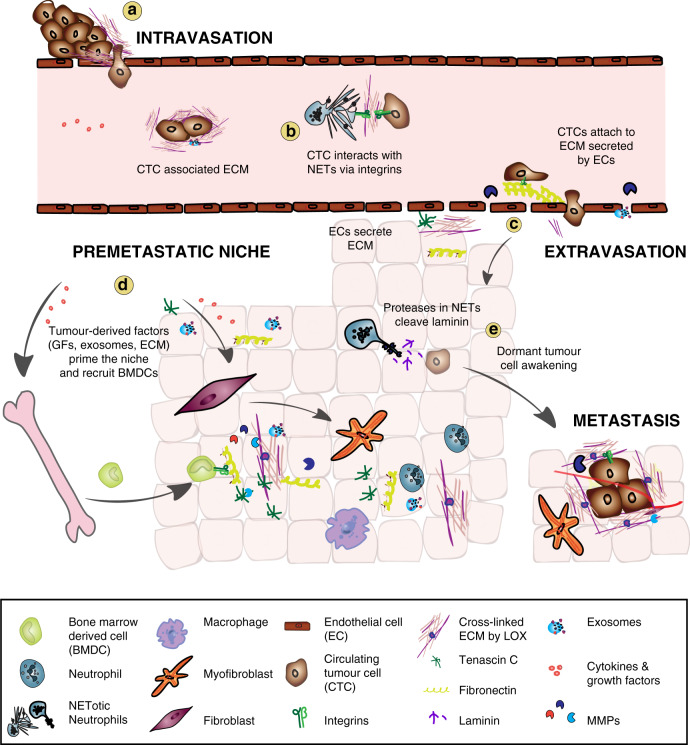
ECM remodelling in the metastatic cascade. **a** Angiogenesis and high MMP activity at the primary tumour site lead to a disrupted vasculature that allows tumour cells to intravasate and enter the circulation. Circulating tumour cells (CTCs) may secrete ECM that protects them from immune surveillance. **b** CTCs may connect via matrix-like interactions with neutrophil extracellular traps (NETs) and NETotic neutrophils using integrins that are expressed on CTCs and neutrophils. **c** Endothelial cells (EC) deposit and assemble fibrillar fibronectin that promotes the attachment of CTCs to the endothelial wall at distant organs. Increased MMP activity creates a leaky vasculature allowing CTCs to extravasate into the surrounding tissue. ECs also remodel the tissue in distant organs and deposit ECM contributing to the establishment of a pre-metastatic niche. **d** Various factors derived from the primary tumour such as growth factors, MMPs, LOX, ECM proteins like fibronectin, and exosomes, create a pre-metastatic niche at a distant site to prime the new tissue for metastasis. Stromal cells in the pre-metastatic niche are activated by tumour-derived factors and myofibroblasts remodel the ECM, for example, by the deposition of fibronectin, tenascin C, osteopontin, and versican depending on tissue context. Bone marrow-derived cells (BMDCs) are recruited to the pre-metastatic niche, attach to the remodelled ECM via integrins and contribute to further ECM remodelling in preparation for the arrival of disseminated tumour cells. **e **CTCs that extravasate through the disrupted vasculature into the distant tissue may become dormant. Proteases expressed in NETs including neutrophil elastase and MMP-9 cleave laminin, generating a specific matrikine that can awaken dormant tumour cells. Together, these ECM remodelling processes support the formation of metastasis.

Crucial for cancer therapy is the prevention and elimination of cancer metastasis, which accounts for the majority of cancer-related deaths. Extensive research during the last decade has unravelled the significance of ECM remodelling at each stage of metastasis development, from surviving in circulation, to forming the pre-metastatic and metastatic niches (Fig. 4).

### ECM remodelling in circulation

As discussed above, various ECM remodelling events support the migration and invasion of tumour cells that eventually will be able to enter circulation. Surprisingly, circulating tumour cells (CTCs) in the blood upregulate expression of common stroma-derived ECM proteins, such as collagens *(Col1a2, Col3a1)*, TIMP-2, the proteoglycan decorin (*Dcn)*, the glycoprotein osteonectin *(Sparc)*, and fibronectin, as revealed by single-cell RNA-sequencing of pancreatic CTCs (Fig. 4a)^202,203^. Yet, it needs to be determined if CTCs actively secrete ECM and which role these ECM components may play in supporting CTCs. Potentially, CTC-secreted ECM could increase autocrine survival signalling of CTCs, protect them from immune cell clearance, similar to platelets surrounding CTCs^204^ or promote the formation of CTC clusters for efficient metastatic colonisation^205^. A recent study showed that metastasis is increased by CTCs when captured by neutrophil extracellular traps (NETs) through interaction with integrin-β1, expressed by both CTCs and NETs (Fig. 4b)^206^. The CTC-secreted ECM components thereby may serve as bridging molecules between NETs and CTCs.

### ECM remodelling in the pre-metastatic niche

Potential sites of metastasis possess organ-specific microenvironments, which are very different from that of the primary tumour. The primary tumour triggers the formation of a favourable microenvironment at the distant tissue, which includes ECM remodelling, even before the presence of metastatic tumour cells, creating a so-called pre-metastatic niche^207^. Of note, at the metastatic site, ECM composition and remodelling processes are distinct from both the primary tumour and the healthy tissue^208–210^ (reviewed in Haye and Erler^211^). The most common alteration of the ECM in the primary tumour environment is increased collagen deposition. In contrast, mainly fibronectin is involved in the formation of the pre-metastatic niche along with glycoproteins and proteoglycans such as tenascin C, osteopontin and versican^207,211^.

Primary tumour-derived factors activate stromal cells in the pre-metastatic site to secrete new ECM molecules or remodel and modify the ECM directly (Fig. 4d). Citrullination, which is the conversion of arginine residues into the non-coding amino acid citrulline, affects the electrostatic charge and folding conformation of fibronectin and collagens and thereby their cell-adhesive properties. Citrullination is mediated by peptidyl-arginine deiminases (PADs), that may be released into the extracellular space on neutrophil extracellular traps during inflammation^212–214^ and potentially also during pre-metastatic niche establishment. However, PAD4 secreted by colorectal cancer cells induces citrullination of collagen I in the liver^215^, which promotes the adhesion of disseminated tumour cells to the liver tissue. In the bone, tumour-derived factors create either an osteolytic (bone-degrading) or osteoplastic (bone-forming) pre-metastatic environment. The more common osteolytic metastasis occurs often in breast cancers as well as other cancers types, where bone homoeostasis is modulated by promoting the activity of bone-resorbing osteoclasts by factors such as IL-6, EGF-like growth factors, TGF-α, NF-κB ligand (RANKL) but also through tumour-derived LOX^216–219^. Moreover, ECM degradation is essential for releasing these soluble factors, such as the liberation of EGF-like growth factors and TGF-α in the primary tumour is mediated by MMP-1 and ADAMTS1^219^. Osteolytic bone metastasis includes the degradation of collagen I, the most common ECM component in the bone. Collagens can also undergo spontaneous non-enzymatic isomerization. Newly synthesised collagen is non-isomerised and is increased in bone metastasis to compensate for the high ECM turnover. Thus, the non-isomerised C-telopeptide of collagen I (α-CTX-I) can be used as a biomarker for bone metastasis from breast and prostate cancers instead of the isomerised form (β-CTX-I)^220^. In contrast, growth factors produced by prostate cancer cells trigger osteoblast differentiation, creating an osteoblastic lesion in the bone for tumour colonisation^221,222^. However, osteolytic events may also occur in osteoblastic bone metastasis since bone matrix-bound growth factors, which are released and activated upon ECM degradation, may be important for metastatic cell proliferation.

Signals from the primary tumour also stimulate the recruitment and adhesion of specific bone marrow-derived cells (e.g., macrophages, neutrophils and myeloid progenitor cells) to the pre-metastatic niche (Fig. 4d)^223,224^. For example, hypoxia-induced LOX expressed by the primary tumour acts at the metastatic site, where it cross-links collagen IV, which provides the adhesion for bone marrow-derived cells, as shown in breast cancer^225^. With their potent ECM remodelling capabilities, bone marrow-derived cells, in turn, promote colonisation of the metastatic tumour cells^226,227^. In another example, colorectal cancer metastasises preferentially to the liver, which correlates with high systemic levels of the MMP inhibitor, TIMP-1. Although TIMP-1 inhibits ECM turnover, it has been implicated in the recruitment of neutrophils to the liver, which then remodel the tissue establishing a tumour-permissive niche^228^. CTCs may contribute to upregulated TIMP-1 levels^229^.

Cancer cell-derived MMPs, such as MMP-3 and MMP-10, induce vascular disruption at the pre-metastatic site supporting the extravasation of CTCs and enhance ECM turnover, which opens up the way for infiltrating bone marrow-derived cells and tumour cells^230^. Endothelial cells also deposit ECM components at the metastatic tissue. Mature endothelial cells can suppress breast cancer growth and mediate their quiescence through the secretion of thrombospondin-1. However, an MMP-mediated disrupted vasculature at the metastatic site^230^ results in the induction of newly sprouting endothelial tip cells that produce TGF-β1 and ECM molecules involved in pre-metastatic niche development, such as periostin, tenascin, versican and fibronectin^231^.

Likely, many of the tumour-derived factors acting in distant organs to prime the metastatic site for tumour cell colonisation may be transported in the form of exosomes. In their lumens, exosomes transport various proteins, such as proteases, and nucleic acids, mainly miRNAs, which are effective at the target site. The composition of exosomes is heterogeneous depending on cell type, and functional or developmental state of the cell and subject of intense research efforts^232,233^.

There is now mounting evidence that metastases of certain cancers show a tissue specificity^234^, suggesting that organ-specific tissue environments including their particular ECM may influence the growth of metastasis^235^. Surprisingly, tumour-derived exosomes may mediate the non-random pattern of metastasis and promote pre-metastatic niche development. Specific groups of integrins expressed on tumour-derived exosomes dictate their adhesion in a particular organ and thereby may determine the site of the future metastasis. Integrins α6β4 and α6β1 guide exosomes to the lung to target fibroblasts and epithelial cells, while exosomal integrin αvβ5 determines their transport to the liver for the uptake by Kupffer cells^236^. To date, it is unclear to what extent this role depends on ECM remodelling in the target tissue and which specific alterations in the ECM cause the organotropic exosome distribution. However, these tumour-derived exosomes create pro-inflammatory and fibrotic environments that recruit bone marrow-derived cells, further promoting ECM remodelling to host metastatic cells. Pancreatic cancer-derived exosomes contain macrophage migration inhibitory factor (MIF) that induces TGF-β secretion by Kupffer cells in the liver, which stimulates an enhanced fibronectin deposition by neighbouring hepatic stellate cells. The resulting fibrotic environment recruits bone marrow-derived macrophages^237^ and ultimately fosters liver metastasis. These studies link the well-known preferred organotropic metastasis pattern of different tumour entities with the novel concept of pre-metastatic niche establishment through organ-specific, exosome-mediated intercellular communication cascades.

### ECM remodelling in the metastatic niche

To form metastases, CTCs need to extravasate from the circulation into the new tissue, a process that is dependent on ECM remodelling. Endothelial cells in the liver of colorectal cancer patients deposit and assemble fibrillar fibronectin that allows the attachment of CTCs to the vascular lumen, induces integrin-dependent focal adhesion and eventually the extravasation into the liver tissue (Fig. 4c)^238^.

After colonisation, stromal and metastatic cells both contribute to create a metastatic niche and actively change the ECM to promote metastastic growth. Interestingly, ECM remodelling of the metastatic niche is different for the same tumour at different sites, indicating tissue tropism. Unique combinations of matrisomal and ECM-associated proteins are deposited by stromal and cancer cells, respectively, at brain, lung, liver and bone metastases in a breast cancer mouse model^239^. Glycoproteins, such as osteopontin, periostin and tenascin C are deposited either by infiltrating tumour cells or by stromal cells at the metastatic site and are important for the colonisation of tumour cells by inducing stemness-like signalling pathways^240–242^. Breast cancer cells in the lung produce tenascin C, which triggers Notch and Wnt signalling^240^. Periostin deposition by activated lung fibroblasts also increases Wnt signalling in metastatic breast cancer cells. The expression of osteopontin and tenascin C depends on the c-Jun transcription factor. Interestingly, chemotherapeutic treatment can activate the c-Jun N-terminal kinase (JNK) pathway and the deposition of these glycoproteins, fuelling metastatic progression^243^. CTCs exhibit mesenchymal characteristics, whereas primary and established metastatic tumour cells show more epithelial phenotypes^242^. Versican deposition by bone marrow-derived cells promotes metastasis by inducing the transition from mesenchymal to epithelial phenotype in metastatic cells^244^, suggesting that this proteoglycan may promote the establishment and progression of metastasis^242^.

Changes in the ECM may also be essential to reactivate disseminated but dormant tumour cells. Recently, matrikines have been associated with awakening dormant tumour cells and promoting metastasis^245^. In an inflammatory environment, neutrophil extracellular traps bind to extracellular laminin and, owing to the presence of laminin-degrading proteases like neutrophil elastase and MMP-9, induce laminin degradation. This proteolytic processing results in the exposure of a specific laminin epitope. This matrikine triggers an integrin-mediated signalling cascade, which leads to the awakening of dormant cancer cells at sites of metastasis and their subsequent proliferation (Fig. 4e)^245^. This study shows the importance of the complex interplay of ECM, immune and tumour cells, with direct implications on metastasis formation.

Further detailed research is needed to uncover the drivers of pre-metastatic niche development and to unravel the intrinsic factors that trigger metastatic ECM remodelling, which may lead to promising therapeutic interventions for the prevention and elimination of cancer metastasis.

## Conclusions and perspectives

Changes in ECM abundance, composition, architecture and the resulting cell-ECM interactions have fundamental consequences on cell and tissue functions. These processes are spatially and temporally regulated to preserve the homoeostasis of tissues and involve the interplay of different cell types. Aberrations in ECM remodelling play a profound role in the development and progression of cancer. Due to their expression in specific pathologies, these ECM alterations can be used as biomarkers and therapeutic targets, for example, by using nanobody technology, which can selectively detect these alterations in vivo and guide therapeutics to the specific location^246^. Several recent examples of ECM-targeted immunotherapies have shown promising results in pre-clinical models^247–250^. However, detailed ECM compositions in various disease contexts are still unclear as are the specific targets of different proteases. More clinical trials focused on specifically targeting the ECM remodelling events are needed giving its importance in disease (Box 2 and Table 2). Different immune cells significantly contribute to changes in the ECM and are also functionally affected by these changes. In recent years, novel immunotherapies promised wide-ranging improvements of treatment for cancer patients. However, many tumours do not respond to these therapies, which may partly due to the immune infiltration barriers caused by the ECM. Targeting these ECM barriers could result in more effective immunotherapy^134^.

**Table 2:** Clinical trials of cancer therapies targeting ECM and ECM-associated molecules.

Target	Agent	Effect on ECM	Cancer type	Status	Ref.
MMP-2 and MMP-9	COL-3 (Incyclinide)	Inhibit MMP activity and expression leading to reduced ECM remodelling and angiogenesis	Refractory metastatic cancer	Completed phase I	NCT00001683^a^
MMP-9	Andecaliximab (anti-MMP-9 mAb) in combination therapy	Inhibit MMP-9 remodelling of ECM	Advanced gastric or gastroesophageal junction adenocarcinoma	Failed phase III	NCT02545504
	(GS) 5745 (anti-MMP-9 mAb) in combination with bevacizumab (anti-VEGF)	Inhibit angiogenesis via inhibition of MMP-9 remodelling of ECM	Recurrent glioblastoma	Ongoing phase I	NCT03631836
MT1-MMP	BT1718 (drug conjugate)	Induce cell death in MT1-MMP expressing cells	Advanced solid tumours	Ongoing phase I/II	NCT03486730
ADAM10 and ADAM17	INCB007839 (aderbasib) in combination with rituximab (anti-CD20)	Decrease cell surface protein degradation and prevent oncogenic signalling from EGFR and Notch pathways	Diffuse large B-cell Non-Hodgkin lymphoma	Completed phase II	NCT02141451
	INCB007839 (aderbasib)	Prevents NLGN3 release into the tumour microenvironment to limit tumour growth	Children with recurrent/progressive high-grade gliomas	Ongoing phase I	NCT04295759
LOX and LOXLs	Tetrathiomolybdate (nonspecific copper chelator)	Chelate copper, which lowers serum LOXL2 concentrations^275^	Moderate to high risk primary breast cancer	Ongoing phase II	NCT00195091
	Tetrathiomolybdate in combination with Carboplatin/Pemetrexed	Chelate copper, which lowers serum LOXL2 concentrations	Metastatic non-small-cell lung cancer	Completed phase I	NCT01837329
	AB0024 (anti-LOXL2 mAb)	Inhibit LOXL2 activity resulting in decreased collagen cross-linking and decreased fibroblast activation	Advanced solid tumours	Completed phase I	NCT01323933
Heparanase	SST0001 (Ronespartat, a chemically modified heparin)	Inhibit heparanase activity resulting in decreased angiogenesis	Advanced, heavily pretreated refractory multiple myeloma	Completed phase I	NCT01764880
HA	PEGPH20 (recombinant human hyaluronidase) in combination therapy	Degrade hyaluronan	Stage IV untreated pancreatic ductal adenocarcinoma (PDA)	Terminated phase III	NCT02715804^a^
TGF-β	Losartan (angiotensin II receptor agonist) in combination therapy	Decrease TGF-β expression, which leads to reduced pro-tumourigenic ECM remodelling including secretion of collagen I	Locally advanced pancreatic cancer	Ongoing phase II	NCT01821729
	Fresolimumab (GC1008, anti-TGF-β mAb) in combination therapy	Prevent fibrosis associated with radiation therapy in breast cancer	Metastatic breast cancer	Completed phase II	NCT01401062
	Fresolimumab (anti-TGF-β mAb) in combination therapy	Prevent fibrosis associated with radiation therapy in early stage non-small-cell lung carcinoma	Stage IA-IB non-small-cell lung cancer	Ongoing phase I/II	NCT02581787

For clinical trials please visit ClinicalTrials.gov.

^a^Representative trial for molecules that have had multiple trials and no ongoing trial.

Progress in investigating cells in their complex intervening three-dimensional networks has resulted in the development of various organoid models that may provide better in vitro tools to investigate the effects of specific matrix perturbations on complex cell–cell and cell–matrix interactions^251^. However, conclusions drawn from in vitro studies using Matrigel and other matrices should be handled with care since they are highly dependent on experimental conditions and lack the complexity of ECM composition and organisation in vivo. Optimised proteomics analysis now provides tools to study the entire ECM of tissues in various contexts^252^. An interesting research direction has also been opened up with the popularisation of novel single-cell technologies, which shed light on the cellular context of ECM remodelling and cell–ECM interactions at a single and sub-cellular level. Advanced tissue imaging approaches promise to give high-resolution insights into the spatial and temporal organisation of these cell-ECM interactions. Novel technologies like multiplexed ion beam imaging (MIBI) can provide a quantitative map of the proteomic landscape of single cells in primary tumours and metastases^253^. With these advanced technologies, biomedical research can take the next step in uncovering the comprehensive interactions of individual cells within their environment, which can lead to the development of more effective ECM-targeted therapies.

Box 2 Therapies targeting ECM remodelling in cancer and metastasis ECM stiffness increases cancer cell proliferation and survival, and induces immunosuppression, even during therapy^286^. Tumourigenic ECM modifications and stiffness can also influence responses to anticancer agents in vitro^287^. Therapies that resolve such stiffness may allow for improved drug penetration to the tumour cells. On the other hand, ECM degradation has been linked to cancer cell migration, invasion and induction of angiogenesis^288^. As such, a regulated approach for the administration of therapies for either ECM stiffness or ECM degradation is critical for effectively controlling the effects of either without inducing the other.

To date, several promising therapies targeting the ECM (Table 2) have not proved effective in the clinic. Examples include targeting fibroblast activation protein-α (FAP), a membrane-bound serine protease expressed in the tumour stroma, but not in normal tissue. Although initial clinical trials resulted in poor outcomes, different strategies for targeting CAFs have been adopted in the ongoing clinical trials including the use of a variant of interleukin-2 as monotherapy or in combination with cetuximab or trastuzumab for head and neck and breast cancers^289^.

Inhibiting LOX-mediated collagen cross-linking, which is necessary for the development of ECM stiffness by targeting the β1-containing integrins that bind to collagen I fibres has been proposed for the prevention of cancer pre-metastatic niche formation and for the treatment of cancer metastasis. However, because β1-integrins interact with multiple ECM components in addition to collagen I, β1-integrin antagonists may have unintended effects^121^. Another therapeutic approach being explored for targeting ECM remodelling with promising but so far limited effect is targeting the TGF-β pathway, known for its role in inducing increased collagen I deposition and decreased degradation when activated. This pathway is being targeted at the ligand, receptor-ligand and intracellular levels with trabedersen, an antisense oligonucleotide that targets TGF-β2; fresolimumab, an anti-TGF-β monoclonal antibody that prevents ligand-receptor interaction; and galunisertib, a small molecule inhibitor of TGF-βRI that prevents signal transduction^290^. MMPIs such as marimastat, incyclinide, etc., that were designed to target multiple MMPs and control ECM degradation, did not improve patient survival due to lack of specificity and severe side effects^289,291^. Studies suggest that targeting individual MMPs at an advanced stage of the disease with monoclonal antibodies such as anti-TIMP-1 monoclonal antibody might improve specificity and patient outcomes as MMPs are significantly increased at advanced stages of cancers^292^. Because the existing therapies seem to be complicated and ineffective, there is a need for more research to improve these therapies and find effective therapies for cancer treatment.
